# Results of Nonoperative Treatment for Symptomatic Tarsal Coalitions

**DOI:** 10.7759/cureus.2944

**Published:** 2018-07-08

**Authors:** Eric Shirley, Radu Gheorghe, Kevin M Neal

**Affiliations:** 1 Orthopaedics, Pediatric Orthopaedic Associates; 2 Orthopaedics, The Hughston Clinic, Jacksonville, USA; 3 Orthopaedics, Nemours Children's Hospital, Jacksonville , USA

**Keywords:** tarsal coalition, talocalcaneal coalition, calcaneonavicular coalition, pediatric foot anomaly

## Abstract

Introduction: Recommendations for the initial treatment (nonoperative measures to surgical excision) of symptomatic tarsal coalitions vary. Because nonoperative outcomes are poorly established, we retrospectively evaluated their success in preventing surgery and achieving pain relief for pediatric patients with symptomatic tarsal coalitions.

Materials and methods: A retrospective study of pediatric patients with symptomatic tarsal coalitions treated at a single institution was undertaken. Clinical notes were examined for treatment methods, response to treatment, and need for additional procedures. A statistical analysis was performed using the chi-square and Mann-Whitney U tests.

Results: Fifty symptomatic tarsal coalitions (mean patient age, 11.4 years; range, 8.1–17.9) were treated with nonoperative measures. Surgery was not required in 79% of calcaneonavicular and 62% of talocalcaneal coalitions. Pain relief was achieved in 53% of 81 nonoperative treatment trials. Continuous immobilization via casting, intermittent immobilization via walking boot, and supportive measures were not significantly different in pain relief (p = 0.35) or preventing surgery (p = 0.62).

Conclusion: Nonoperative treatment methods have the potential to achieve pain relief and prevent or delay surgery for symptomatic tarsal coalitions. However, some families may elect to forgo nonoperative measures knowing that surgery may eventually be required.

## Introduction

**Figure 1:**
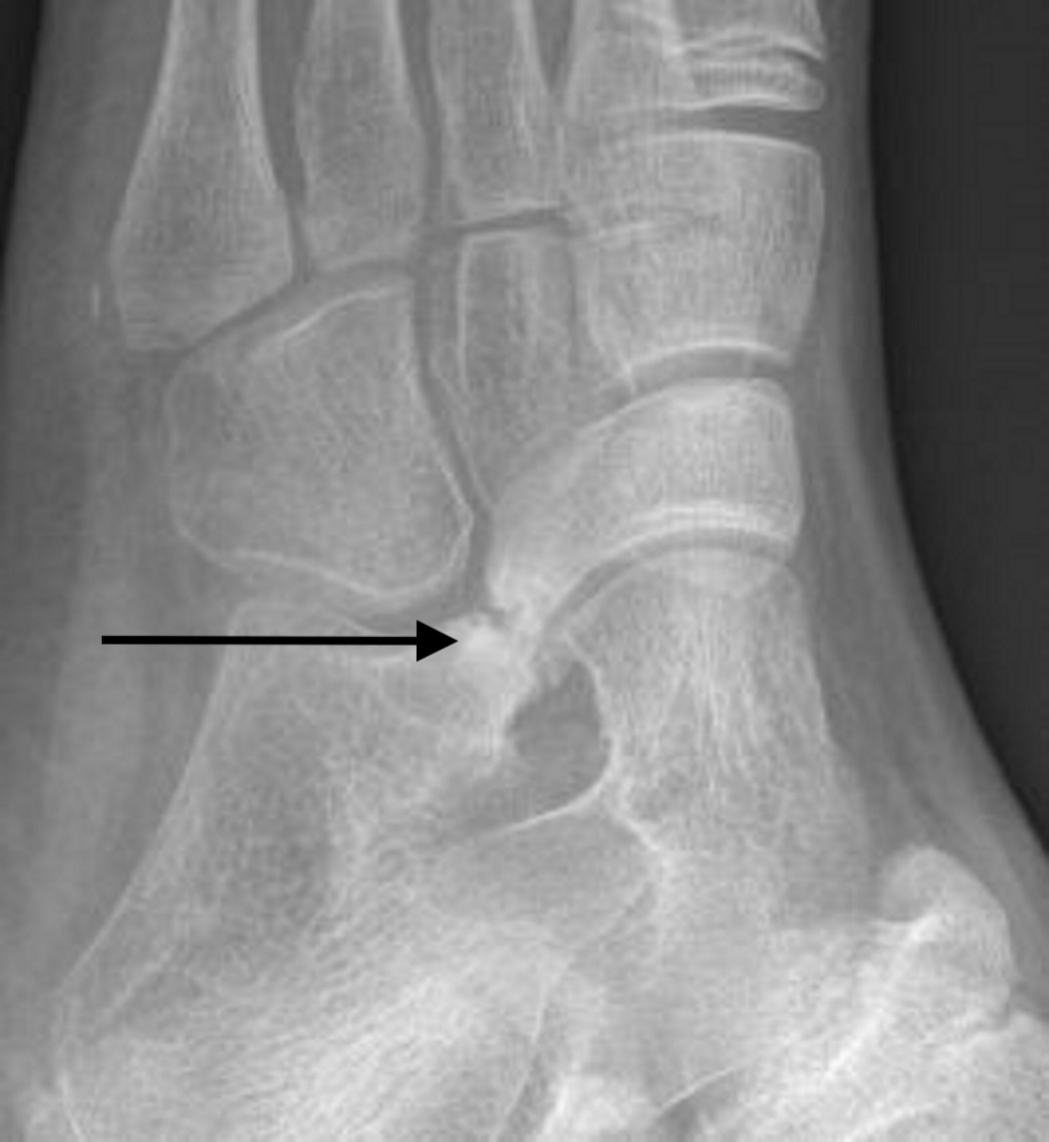
Oblique view of left foot demonstrating calcaneonavicular coalition.

Tarsal coalitions occur in less than 1% of the population [1]. While many patients are asymptomatic, some develop pain, activity limitations, or recurrent ankle sprains [2-4]. The onset of these manifestations often correlates with the ossification of the coalition: between the ages of eight and 12 years for calcaneonavicular (CN) coalitions and 12 and 16 for talocalcaneal (TC) coalitions [5]. Plain radiographs including an oblique view for calcaneonavicular coalitions, as shown in Figure 1, are obtained to confirm the diagnosis. When symptoms occur, nonoperative treatments, such as activity modification, nonsteroidal anti-inflammatory medications, or over-the-counter longitudinal arch supports, may be initiated [6-7]. Other nonoperative options include the University of California Biomechanical Laboratory (UCBL) orthosis and immobilization in a walking boot or short leg cast. Surgical options include resection for both types of coalitions and consideration of fusion for large TC coalitions.

Which treatment to initiate first for a symptomatic coalition is somewhat controversial. Jayakumar and Cowell suggested that resection is preferred for CN coalitions, as the procedure will better achieve the goal of restoring normal foot function [8]. Mubarak et al. also recommend surgery as the first-line treatment for CN coalitions due to poor results at their institution with cast treatment [3]. Similarly, Murphy and Mubarak [9] recommended surgery for TC coalitions, suggesting that surgery will prevent further symptoms by addressing the underlying pathology, which is not achieved with casting [2]. Most other authors recommend nonoperative measures for both symptomatic CN [10-13] and TC [7-8,10-12,14-16] coalitions.

However, the efficacy of nonoperative measures for symptomatic tarsal coalitions is poorly established. While the success rate of 33% using cast immobilization for symptomatic TC coalitions reported by Jayakumar and Cowell [8] is often cited [7,13-14,17], the number of patients, ages, and follow-up were not reported. While later studies have included further supporting details for nonoperative treatment, no study to date has focused primarily on the outcomes of nonoperative treatment for symptomatic coalitions.

Our objective was to retrospectively evaluate the success of nonoperative measures in preventing surgery and achieving pain relief for pediatric patients with symptomatic tarsal coalitions. We also sought to identify factors associated with the prevention of surgery via successful nonoperative treatments.

## Materials and methods

After obtaining institutional review board (IRB) approval, International Classification of Diseases-9 (ICD-9) [18] codes were used to identify patients <18 years of age who were treated at a single institution for tarsal coalitions over a six-year period. Clinic notes were examined to identify patient age, gender, type of coalition, method of diagnosis, and symptoms. Specific treatments and the outcomes of each were recorded.

Nonoperative treatment methods were classified as continuous immobilization (short leg cast), intermittent/removable immobilization (walking boot, short leg splint, ankle foot, or supramalleolar orthosis, and UCBL orthosis), and supportive measures (shoe inserts and physical therapy). Outcome measures for nonoperative treatment methods were pain relief and prevention of surgery (defined as the treated coalition not requiring surgery during clinical follow-up). The outcomes of the various nonoperative methods and coalition types were compared statistically using the chi-square and Mann-Whitney U tests. Significance was set at p < 0.05. Analyses were also descriptive based on the distribution of results.

## Results

Seventy-one symptomatic tarsal coalitions in 70 patients underwent treatment during the study period. Forty-seven coalitions were diagnosed via radiographs alone while 24 underwent computed tomography (CT) or magnetic resonance imaging (MRI). Thirty percent (21) of the 71 coalitions underwent surgery without a trial of nonoperative measures and, thus, were excluded from the analysis.

The other 50 symptomatic coalitions (33 CN, 16 TC, one talonavicular) in 49 patients (mean age 11.4 years, range 8.1–17.9) underwent at least one trial of nonoperative treatment. A synopsis of the results of the nonoperative treatment measures is shown in Table 1. There were a total of 81 trials of nonoperative treatment. Short leg casts and walking boots were applied for a mean 4.3 weeks (range, 3-8 weeks) and 4.4 weeks (range, 3-8 weeks), respectively. UCBL orthoses were worn for a mean 1.5 years (range, 2 months–3 years). Pain relief was achieved with 61% of the trials with supportive measures, 57% with continuous immobilization, and 40% with intermittent immobilization. The rates of pain relief between the three types of interventions were not significantly different (p = 0.35). Surgery was prevented in 75% of coalitions treated with intermittent immobilization, 72% with supportive measures, and 64% with casting. There were no significant differences between the treatment methods in terms of preventing surgery (p = 0.62).

**Table 1:** Results of nonoperative treatment measures UCBL: University of California Biomechanical Laboratory

Type of Treatment	Number of Coalitions	% Pain Relief	% Surgery Prevented
Continuous immobilization (short leg cast)	31	57	64
Intermittent immobilization totals	32	40	75
Walking boot	22	41	68
Short leg splint	1	100	100
Ankle-foot or supramalleolar orthosis	6	50	83
UCBL orthosis	3	0	100
Supportive measure totals	18	61	72
Activity modification, rest, nonsteroidal anti-inflammatory medications	3	0	33
Ankle support (lace-up, brace)	5	80	100
Physical therapy (home or formal)	7	71	86
Shoe inserts	3	67	100
All nonoperative treatments	81	53	70

Pain relief was achieved in 55% of nonoperative trials for CN coalitions and 54% for TC coalitions. Surgery was prevented in 79% (26/33) of the CN coalitions and 62% (10/16) of the TC coalitions. The differences in pain relief (p = 0.86) and preventing surgery (p = 0.39) between the coalition types were not statistically significant. The one talonavicular coalition was treated with three trials of nonoperative treatment over a four-year period. Surgery was not required when the patient was last seen at age 13.5 years.

Surgery was not required in 74% of the 50 coalitions with a mean follow-up of 13.2 months (range, 0.7–88.5) and 62% of 34 coalitions with a mean follow-up of 19.8 months (range, 3.1–88.5). Thirteen coalitions eventually underwent surgery. Nonoperative treatment for coalitions that did not require surgery occurred in younger patients (mean 11.5 years; range, 8.8–17.9 vs. mean 12.5 years; range, 10.5–17.5) and those who underwent more trials of nonoperative treatment (mean, 2.6; range, 1–3 vs. mean 1.8; range, 1–3). These differences in age (p = 0.50) and number of treatment trials (p = 0.51) were not significant.

## Discussion

Much of the literature on the treatment of tarsal coalitions is limited by a primary focus on surgical outcomes [14-15,19-20]. Takakura et al. reported the operative and nonoperative outcomes of 67 TC coalitions in pediatric and adult patients [19]. Cold compresses were used for 12 feet, longitudinal arch support for 10 feet, medial heel wedge for five feet, short leg cast for three feet, and a UCBL orthosis for one foot. Excellent or good results were noted in 68% of the patients, but the outcomes of each intervention type were not reported. Scranton also reported [20] the operative and nonoperative outcomes of 23 symptomatic TC coalitions in pediatric and adult patients. All five feet (ages 35–55) treated nonoperatively achieved a satisfactory result with casting. Kumar et al. evaluated 16 patients who underwent surgical resection of TC coalitions and noted that approximately 1/3rd of the patients had initially achieved relief with nonoperative measures [14]. However, the number of patients who completely responded to nonoperative measures and did not undergo surgery was not reported. Gonzalez and Kumar [15] reported the long-term outcomes of CN resection for 75 feet. Fifteen of the 75 feet underwent initial nonoperative treatment with soft inserts followed by a short leg cast and none achieved permanent relief. Less symptomatic patients treated with shoe inserts or casts and lost to follow-up were excluded.

Other studies on nonoperative treatment for tarsal coalitions are limited by the inclusion of all types of peroneal spastic flatfeet, including those without coalitions [21-22]. Braddock [21] evaluated 56 peroneal spastic flatfeet (ages 10–15 years) treated with manipulation under anesthesia and short leg casting. Of the 10 TC coalitions; 20% (2/10) were pain-free, 60% (6/10) had mild symptoms, and 20% (2/10) had severe symptoms. Of the 12 CN coalitions, 58% (7/12) were pain-free and 42% (5/12) had mild pain after treatment. Blockey [22] evaluated 30 peroneal spastic flatfeet treated with examination under anesthesia, casting in inversion, followed by physical therapy and shoe modifications. Seventy-five percent of the four TC coalitions (ages 14–39) had pain relief at two years and none required surgery. Thirty-three percent of the nine CN coalitions (ages 10–17) had pain relief at two years and 22% (2/9) required surgery.

The lack of established outcomes of nonoperative measures for tarsal coalitions likely contributes to the 30% of symptomatic coalitions in our series that underwent surgery without a trial of nonoperative treatment. Physicians may have recommended surgery as the best option based on their interpretation of the literature or a personal observation of poor results with nonoperative measures. Alternatively, families may have decided not to spend time with a trial of nonoperative treatment knowing that there is a chance that excision at a later date may still be required.

In our series, nonoperative measures produced pain relief in 53% of treatment trials. Pain relief was most successful in coalitions treated with supportive measures rather than immobilization. It should not be assumed that supportive measures are superior for all patients, as these may have been prescribed to those with less severe pain. We also found that 74% of the coalitions in our series treated with nonoperative measures did not require surgery, decreasing further with longer follow-up. While it is reasonable to conclude that the pain relief achieved by nonoperative measures is not always sustained, this decrease in success over time may also be due to a selection bias with the patients returning for follow-up, to discuss surgery while those who did not return had less pain and chose not to return. 

While surgery was more often prevented in CN coalitions, younger patients, and those with more nonoperative treatment attempts, none of these factors were significant and thus further study is needed to confirm these observations. The possibility of decreased success in older patients may be the result of additional changes in foot biomechanics as the coalition further ossifies. Similarly, the decreased success in talocalcaneal coalitions may reflect different biomechanics and pain pathophysiology. Lastly, success in patients with more nonoperative treatment attempts may simply be a result of these patients having a stronger desire to avoid surgery for timing, cost, or other reasons.

Based on these findings, we believe that a trial of nonoperative treatment is a reasonable initial approach to the symptomatic coalition in pediatric patients. Because short leg casting was not significantly more effective, consideration can be given to less cumbersome options. One approach is to apply a walking boot for six weeks, followed by supportive measures (longitudinal arch supports with ankle strengthening) and gradual return to activities. Patients with less severe pain can initially proceed directly to supportive measures. If activity and lifestyle-limiting symptoms persist, additional nonoperative treatments may be considered, especially in younger patients or those with CN coalitions.

Our study has weaknesses worth noting. We used a subjective measure, pain relief, as an outcome measure due to its feasibility for a retrospective study. It would be helpful to use a validated foot and ankle outcome measure and pain scale in a prospective manner to clarify pain severity and to match treatment success with different pretreatment levels of pain. Our other outcome measure, proceeding with surgery, is also somewhat subjective, as thresholds for proceeding with surgery vary among patients. Our results may also be influenced by compliance, particularly for treatments such as UCBL wear and physical therapy, which is difficult to measure. Lastly, we demonstrated the results of nonoperative measures at early follow-up. Longer follow-up is needed to evaluate if the nonoperative measures prevent or delay the need for surgery.

However, early results are often important to families as well. Factors such as the need to complete a school year, sports season, or insurance plan prior to undergoing surgery are often taken into consideration. In addition, while pain relief and requiring surgery are subjective, these data can still be helpful when educating families about results in other patients. In addition, reporting the outcomes of nonoperative treatment is important, as these measures are not free when considering time, inconvenience, and cost. At some point, multiple nonoperative measures are less cost-effective than surgery and less reasonable to prescribe if they are not effective.

## Conclusions

This is the first study in the literature that focuses solely on the outcomes of nonoperative measures for symptomatic tarsal coalitions. We found that nonoperative treatment attempts have the potential to achieve pain relief and prevent or delay surgery for symptomatic tarsal coalitions in pediatric patients. Additional study is needed to better define the natural history of this condition and the influence of factors such as Achilles contracture on symptoms and symptom relief. Further study is also needed to evaluate the outcomes of nonoperative measures at other centers and the cost-effectiveness of each option.
